# Aldosterone-producing adrenocortical carcinoma with prominent hepatic metastasis diagnosed by liver biopsy: a case report

**DOI:** 10.1186/s12902-016-0083-0

**Published:** 2016-01-16

**Authors:** Kennosuke Ohashi, Takeshi Hayashi, Masaya Sakamoto, Hiroyuki Iuchi, Hirofumi Suzuki, Takanori Ebisawa, Katsuyoshi Tojo, Hironobu Sasano, Kazunori Utsunomiya

**Affiliations:** Division of Diabetes, Metabolism and Endocrinology, Department of Internal Medicine, the Jikei University School of Medicine, 3-25-8 Nishishinbashi, Minato-ku, Tokyo 105-8461 Japan; Department of Pathology, Tohoku University School of Medicine, 2-1 Seiryo-machi, Aoba-ku, Sendai, Miyagi 980-8575 Japan

**Keywords:** Adrenocortical carcinoma, Aldosterone, Metastatic liver cancer, Adrenal 4 binding protein/steroidogenic factor 1, Immunohistochemical staining

## Abstract

**Background:**

Aldosterone-producing adrenocortical carcinoma is a rare malignancy, which is usually diagnosed by histopathological examination of the excised tumor. In inoperable cases, aldosterone-producing ACC diagnosed by immunohistochemical staining of the metastatic tumor for Cytochrome P450 (CYP) 11β has not previously been reported and even in that case staining for adrenocortical-specific adrenal 4 binding protein/steroidogenic factor1 (Ad4BP/SF1) and steroidogenic enzymes has not been reported.

**Case presentation:**

We report the case of a 67-year-old Japanese woman with aldosterone-producing adrenocortical carcinoma. Laboratory findings showed severe hypopotassemia. Endocrinological examination revealed an increased plasma aldosterone concentration and suppressed plasma renin activity. Plasma dehydroepiandrosterone sulfate (DHEA-S) was elevated. Diurnal variation in serum cortisol was lost and administration of 1 mg and 8 mg dexamethasone did not suppress serum cortisol levels. From the 24-h urine collection sample, urine aldosterone and urine cortisol levels were greatly increased. Therefore, autonomous excess production was observed for the three adrenal cortex hormones. Abdominal computed tomography and magnetic resonance imaging showed a right adrenal tumor and a huge liver tumor. Adrenocortical carcinoma with metastatic liver cancer was strongly suggested, however surgery could not be considered due to stage IV disease: the liver tumor was too large and cardiac ultrasonography indicated that her cardiac function was poor. Therefore, a liver biopsy was taken to properly determine the diagnosis. Immunohistochemical stains for Ad4BP/SF1 and steroidogenic enzymes were positive. Ad4BP/SF-1 was originally identified as a steroidogenic, tissue-specific transcription factor implicated in the expression of the steroidogenic *CYP* gene encoding cytochrome P450s. Hence we could diagnose the patient as having adrenocortical carcinoma with metastatic liver cancer.

**Conclusion:**

This rare case had severe hypopotassemia accompanied with not only increased cortisol and DHEA-S but also aldosterone. We reached the diagnosis of adrenocortical carcinoma with metastatic liver cancer based on positive immunohistochemical staining of Ad4BP/SF1 in the liver biopsy specimen. We have reported the first case of aldosterone-producing adrenocortical carcinoma diagnosed solely by immunohistochemical staining for adrenocortical-specific Ad4BP/SF1 and steroidogenic enzymes in a metastatic liver tumor.

## Background

Adrenocortical carcinoma (ACC) is a rare malignancy with an incidence of 1–2 per million people. ACC follows a heterogeneous clinical course and a variable but generally poor prognosis [1–4]. Approximately 60 % of ACCs are hormonally active, and glucocorticoids and/or androgens are the steroids that are frequently over-secreted. A rapidly progressive Cushing’s syndrome with or without virilization is the most frequent manifestation; estrogen or mineralocorticoid excess occurs in 10 % or fewer cases [1–3, 5]. ACC is usually diagnosed by histopathological examination of the excised tumor. In inoperable cases, aldosterone-producing ACC diagnosed by immunohistochemical staining of the metastatic tumor for Cytochrome P450 (CYP) 11β has not previously been reported and even in that case staining for adrenocortical-specific adrenal 4 binding protein/steroidogenic factor1 (Ad4BP/SF1) and steroidogenic enzymes has not been reported.

Here we report the first case of aldosterone-producing ACC diagnosed by immunohistochemical staining in only the metastatic tumor to detect both Ad4BP/SF1 and steroidogenic enzymes.

## Case presentation

A 67-year-old woman with a history of diabetes mellitus and hypertension was referred to our hospital for evaluation of hypopotassemia, a right adrenal tumor and a huge liver tumor. She complained of dizziness and weight loss. We suspected that she had ACC and metastatic liver cancer. Physical examination showed thin skin, hirsutism, hepatomegaly and mild leg edema. She did not have a cushingoid appearance with manifestations such as a moon face, central obesity and buffalo hump.

Laboratory findings (Table 1) showed severe hypopotassemia (K: 1.2 mmol/l), leukocytosis and liver damage. Carcinoembryonic antigen (CEA), carbohydrate antigen 19–9 (CA19-9) and protein induced by vitamin K absence or antagonist-II (PIVKA-II)—which is a hepatocellular carcinoma marker—were elevated.

**Table 1:** Laboratory findings

Urine		Biochemistry	
Protein	(2+)	AST	107 IU/l
Glucose	(+)	ALT	68 IU/l
Ketone	(−)	LDH	1105 IU/l
U-UN	0.119 mg/dl	ChE	3479 IU/l
U-Cr	11.3 mg/dl	T-Bil	1.1 mg/dl
U-Na	28 mmol/l	ALP	636 lU/l
U-K	15.3 mmol/l	γ-GT	344 IU/l
U-Cl	21 mmol/l	TP	6.6 g/dl
		Alb	4.0 g/dl
Blood cell count		UN	24 mg/dl
WBC	16300/μl	Cr	0.8 mg/dl
Neutrophil	84.6 %	UA	4.4 mg/dl
Lymphocyte	9.7 %	Na	145 mmol/l
Monocyte	5.5 %	K	1.2 mmol/l
Eosinophil	0.1 %	Cl	80 mmol/l
Basophil	0.1 %	Ca	8.8 mg/dl
RBC	4.59 × 10^6^/μl	Plasma glucose	159 mg/dl
Hemoglobin	14.4 g/dl	HbA1c	6.0 %
Hematocrit	43.5 %	CRP	6.2 mg/dl
Platelet	36.7 × 10^4^/μl		
Tumor markers			
CEA	17.1 ng/ml (5.8>)	AFP	4 ng/ml (10>)
CA19-9	294 U/ml (37>)	PIVKAII	183 mAU/ml (40>)

Endocrinological examination (Table 2) revealed an increased plasma aldosterone concentration (PAC: 2040.0 pg/ml) and suppressed plasma renin activity (PRA: 0.3 ng/ml/h). Plasma DHEA-S was elevated (294 μg/dl). Diurnal variation in serum cortisol was lost. Fasting plasma levels of adrenocorticotropic hormone (ACTH) and cortisol were less than 2.1 pg/ml (7.2–63.3) and 27.7 μg/dl (4.0–18.2), respectively, and administration of 1 mg and 8 mg dexamethasone did not suppress serum cortisol levels. In addition, 24-h urine was collected, and the urinary aldosterone level was 230.0 μg/day (>10) and the urinary cortisol level was 477.0 μg/day (11.2–80.3).

**Table 2:** Endocrinological examination

			Normal range
Hormonal profile			
Serum ACTH (pg/ml)		< 2.1	7.2–63.3
Serum cortisol (μg/dl)		27.7	4.0–18.3
Plasma renin activity (ng/ml/hr)		0.3	0.3–2.9
Plasma aldosterone concentration (pg/ml)		2040	140–1030
Serum DHEA-S (μg/dl)		294	12–133
Serum 11-OHCS (μg/dl)		566.0	7.0–23.0
Urinary free cortisol (μg/24 hr)		477.0	11.2–80.3
Urinary free aldosterone (μg/24 hr)		230.0	< 10
Diurnal variation of plasma ACTH, cortisol levels			
	9:00	16:00	23:00
Serum ACTH (pg/ml)	< 2.1	< 2.1	< 2.1
Serum cortisol (μg/dl)	43.6	47.7	42.6
Dexamethasone suppression test		1 mg	8 mg
Serum ACTH (pg/ml)		< 2.1	< 2.1
Serum cortisol (μg/dl)		36.0	39.1

**Fig. 1:**
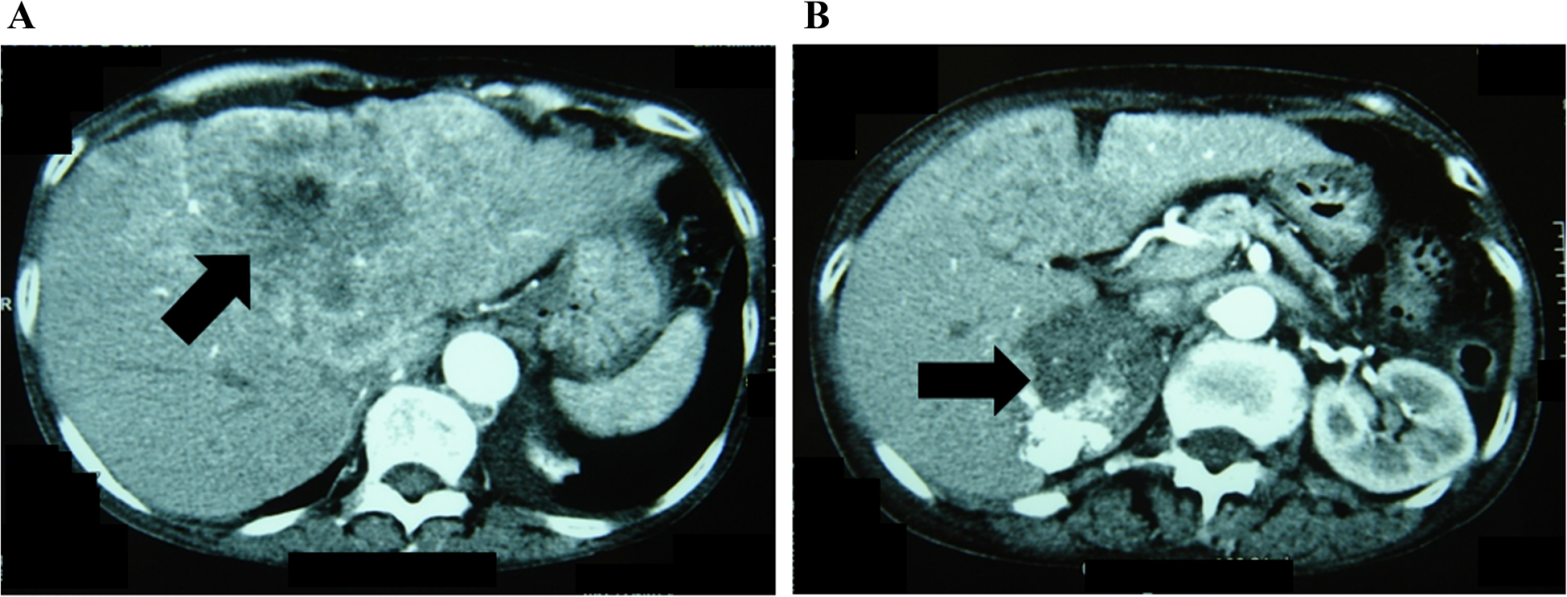
Abdominal dynamic computed tomography. Abdominal dynamic computed tomography shows a huge liver tumor (14 cm in diameter) and right adrenal tumor (6 cm in diameter). **a** slice of liver tumor; **b** slice of right adrenal tumor

Autonomous excess production was demonstrated for the three adrenal cortex hormones (aldosterone, cortisol and DHEA-S). Abdominal computed tomography (CT) showed an internal heterogeneous right adrenal tumor (6 cm in diameter) with calcification as well as a huge liver tumor (14 cm in diameter) with internal necrosis (Fig. 1).

**Fig. 2:**
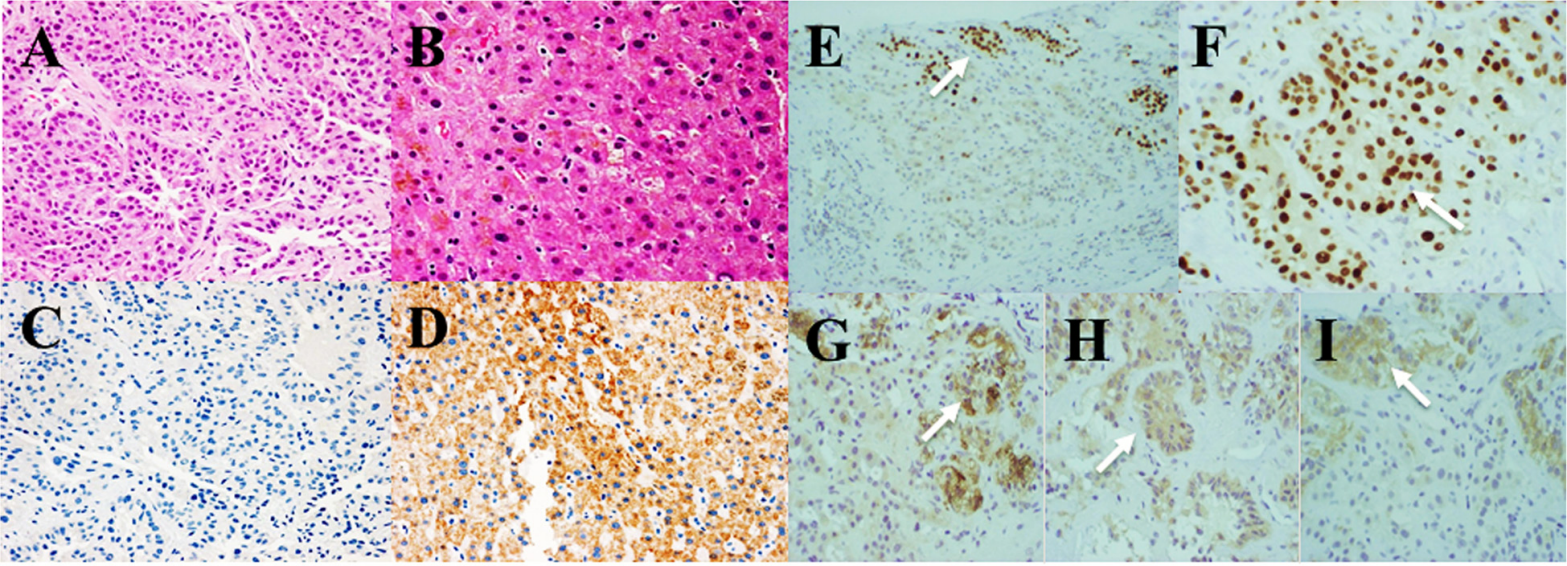
Histopathological diagnosis by examination of liver biopsy specimen. **a** Tumor cells show large conspicuous nuclei, nuclear atypicality, and acidophilic cytoplasm. Cells had a cord-like architectural pattern and alveolar structure (HE staining, original magnification × 400); **b** Normal hepatocytes (HE, ×400); **c** These tumor cells are undyed; **d** Normal hepatocytes became stained (C, D: Hepatocyte paraffin 1 staining, ×400); **e**-**i** Immunohistochemical stainings showed positive reactivity (E: Ad4BP/SF1, ×40; F: Ad4BP/SF1, ×400; G: 3β-HSD, ×40; H: P450c21, ×40; I: Inhibinα, ×40). Black arrow: Ad4BP/SF1 was stained in a nucleus. White arrow: steroidogenic enzymes was stained in cytoplasm

**Fig. 3:**
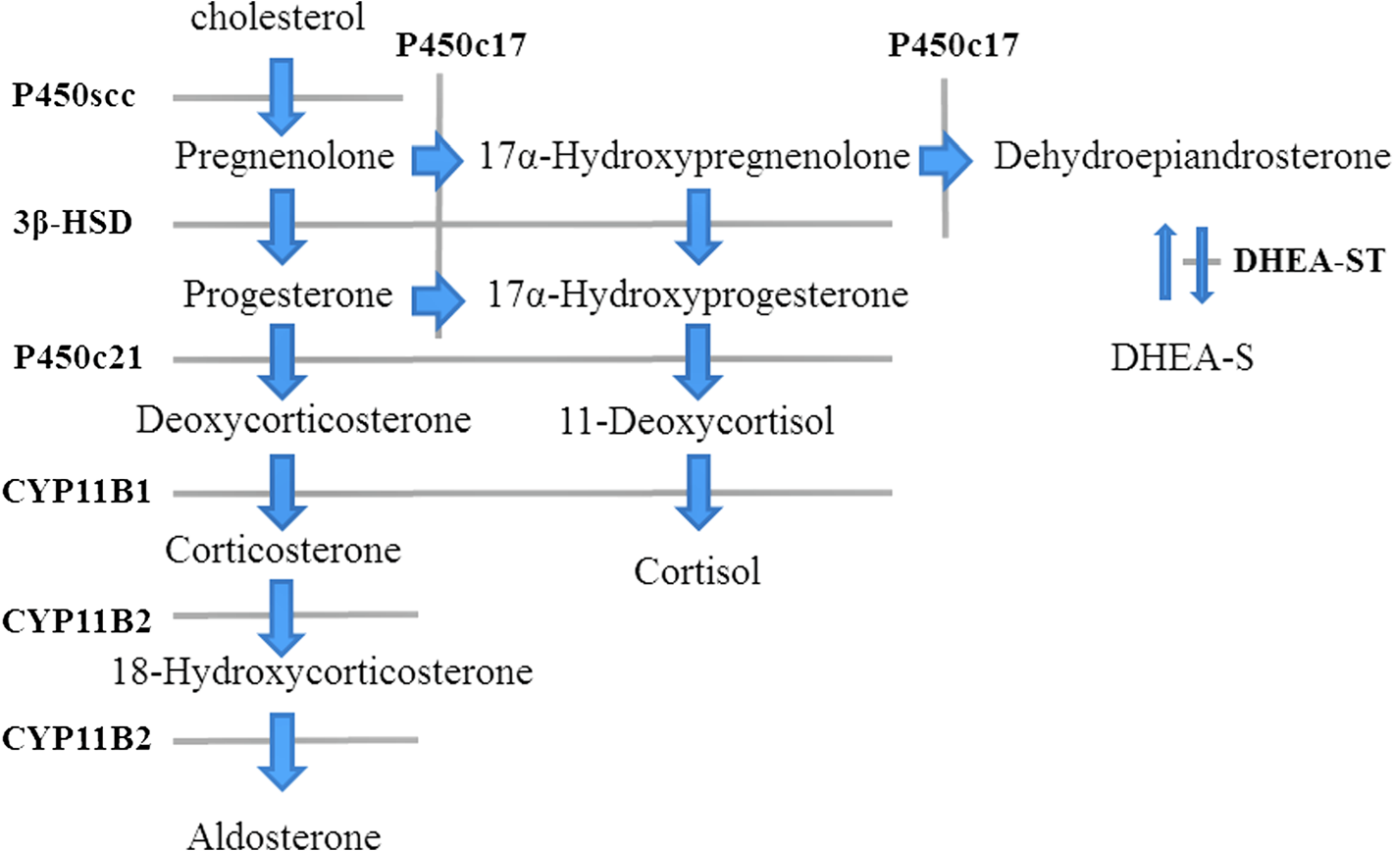
Steroid hormone biosynthesis pathway. We applied immunohistochemical staining for P450scc, 3β-HSD, P450c21, P450c17 and DHEA-ST

As a result of laboratory findings, endocrinological examination and abdominal enhanced CT and MRI, an ACC with widespread metastatic liver cancer was strongly suggested. We wanted to excise the adrenal tumor including the liver tumor for diagnosis and treatment. But this case could not be considered for surgery because of stage IV disease: the liver tumor was too large and cardiac ultrasonography indicated that her cardiac function was poor (ejection fraction: 32 %). Therefore a liver biopsy was taken to determine the diagnosis. At histopathological examination, hematoxylin and eosin (HE) staining indicated a diagnosis of carcinoma. Hepatocyte paraffin 1 staining was negative in this case, so this tumor was not a hepatocellular carcinoma. Immunohistochemical staining for Ad4BP/SF1 and steroidogenic enzymes were positive. P450scc, 3β-HSD, P450c21, P450c17 and DHEA-ST were all positive (Figs. 2 and 3). Therefore, we finally reached a diagnosis of ACC with metastatic liver cancer.

Her prominent hypopotassium values improved (K 3.9 mmol/l) with administration of trilostane (240 mg/day). She was also administered mitotane (1.5 g/day) with dexamethasone.

Reductions in cortisol and aldosterone levels were gradually achieved (cortisol 23.5 μg/dl and PAC 246.0 pg/ml). However, at her request, she was transferred to another hospital where she died 3 months later.

## Discussion

Here we reported the first case of aldosterone-producing ACC diagnosed by immunohistochemical staining in only the metastatic tumor to detect both Ad4BP/SF1 and steroidogenic enzymes.

The present case demonstrated severe hypopotassemia accompanied with not only increased cortisol and DHEA-S but also aldosterone. Approximately 60 % of ACCs are hormonally active, and glucocorticoids and/or androgens are frequently over-secreted. Mineralocorticoid excess is very rare [1–3, 5].

Generally, there is a high suspicious for malignancy if adrenal tumors are larger than 6 cm [6]. Large clinically asymptomatic adrenal masses are treated surgically and then diagnosed by histopathological examination. A microscopic diagnostic score (Weiss score) is the most commonly used tool [7]. Surgery was not considered in this case because disease was classified as stage IV [3]: the liver tumor was too large and cardiac ultrasonography indicated that her cardiac function was poor. Local invasion, tumor extension into the inferior vena cava as well as lymph nodes or other metastases (lung and liver) are often found in advanced ACC [3].

After various medical tests, ACC with widespread metastatic liver cancer was strongly suspected, and a liver biopsy was performed to rule out hepatocellular carcinoma due to increased PIVKA-II score. With HE staining, tumor cells showed large conspicuous nuclei, nuclear atypicality, and acidophilic cytoplasm that had a cord-like architectural pattern and alveolar structure. This tumor was diagnosed as a carcinoma. Hepatocyte paraffin 1 staining was negative, thus indicating that this case did not have hepatocellular carcinoma. We applied immunohistochemical staining for Ad4BP/SF1 and steroidogenic enzymes to distinguish whether cells in this tumor were those of ACC. Ad4BP/SF-1 was originally identified as a steroidogenic, tissue-specific transcription factor implicated in the expression of the steroidogenic *CYP* gene encoding cytochrome P450s [8]. An immunohistochemical evaluation of Ad4BP/SF-1 can aid in this differential diagnosis because nuclear immunoreactivity for this transcription factor is relatively specific to steroid-producing cells. It has been reported that application of Ad4BP/SF-1 immunohistochemistry can greatly contribute to the differential diagnosis of ACC from other malignancies both at primary and metastatic sites [9]. In addition, it has been reported that Ad4BP/SF-1 is a very useful immunohistochemical marker in diagnosing the origin of metastatic sites of ACC [10]. This case had positive immunoreactivity for Ad4BP/SF1. Immunohistochemical staining for steroidogenic enzymes also showed positive reactivity. P450scc, 3β-HSD, P450c21, P450c17 and DHEA-ST were all positive. Therefore we diagnosed that this patient had ACC with metastatic liver cancer.

We identified a case of distant recurrence of ACC after adrenalectomy that was diagnosed by immunohistochemical staining for steroidogenic enzymes in a lung metastatic tumor [11]. An ACC case with pulmonary metastasis diagnosed by a pleural biopsy was also reported [12]. However, in that pulmonary metastasis report, immunohistochemical staining for Ad4BP/SF1 and steroidogenic enzymes was not performed.

We acknowledge several limitations in our report. First, we could not use immunohistochemical staining to examine the original adrenal tumor for Ad4BP/SF1 and steroidogenic enzymes. Second, there is no evidence that this tumor produced aldosterone, because we could not apply immunohistochemical staining for CYP11B1 and CYP11B2 in a metastatic tumor at that time. Despite these limitations, we strongly suspected that these tumors produced aldosterone because PAC increased significantly as a result of endocrinological examination.

## Conclusion

Here, we report a rare case of ACC with severe hypopotassemia accompanied not only increased cortisol and DHEA-S but also aldosterone. We were successful in diagnosing the patient as having ACC with metastatic liver cancer based on positive immunohistochemical staining of metastatic cancer for adrenocortical specific Ad4BP/SF1 and steroidogenic enzymes.

## Consent

Informed consent was obtained from the patient’s family for publication of this case report and any accompanying images.

## Abbreviations

11-OHCS: 11-hydroxycorticosteroid
3β-HSD: 3β-hydroxysteroid dehydrogenase
ACC: Adrenocortical carcinoma
Ad4BP/ SF1: Adrenal 4 binding protein/steroidogenic factor 1
AFP: Alpha fetoprotein
Alb: Albumin
ALP: Alkaline phosphatase
ALT: Alanine aminotransferase
AST: Aspartate aminotransferase
CA19-9: Carbohydrate antigen 19–9
CEA: Carcinoembryonic antigen
ChE: Cholinesterase
CRP: C-reactive protein
CYP: Cytochrome P450
DHEA-ST: dehydroepiandrosterone-sulfotransferase
HbA1c: HemglobinA1c
LDH: Lactase dehydrogenase
P450c17: Cytochrome P450 17
P450c21: Cytochrome P450 21
P450scc: Cytochrome P450 side chain cleavage
PIVKA-II: Protein induced by vitamin K absence or antagonist-II
RBC: Red blood cell
T-Bil: Total bilirubin
TP: Total protein
UA: Uric acid
U-Cr: Urine Creatinine
U-UN: Urine urea nitrogen
WBC: White blood cell
γ-GT: γ-glutamyltranspeptidase
